# Design of novel proliposome formulation for antioxidant peptide, glutathione with enhanced oral bioavailability and stability

**DOI:** 10.1080/10717544.2018.1551441

**Published:** 2019-03-07

**Authors:** Jong Chan Byeon, Sang-Eun Lee, Tae-Hyeon Kim, Jung Bin Ahn, Dong-Hyun Kim, Jin-Seok Choi, Jeong-Sook Park

**Affiliations:** a College of Pharmacy and Institute of Drug Research and Development, Chungnam National University, Daejeon, South Korea;; b Department of Medical Management, Chodang University, Jeollanam-do, South Korea

**Keywords:** Proliposome, peptide drug, glutathione, oral drug delivery system

## Abstract

To develop proliposome formulations to improve the oral bioavailability of l-glutathione (GSH), GSH-loaded proliposomes were prepared using the granule method. Mannitol was selected as an effective excipient to achieve the desired particle size, entrapment efficiency (EE), and solubility for oral delivery of the final formulation. To evaluate the effect of surface charge of proliposomes on the oral bioavailability of GSH, negative (F1–F4) and positive proliposomes (F5–F9) were prepared. Particle size of F1 and F5 was 167.8 ± 0.9 and 175.9 ± 2.0 nm, and zeta potential of F1 and F5 was –8.1 ± 0.7 and 21.1 ± 2.0 mV, respectively. Encapsulation efficiency of F1 and F5 was 58.6% and 54.7%, respectively. Considering their particle size, zeta potential, and EE, the proliposomes F1 and F5 were adopted as the optimal formulations for further experiments. Solid state characterization of the proliposomes confirmed lipid coating on the surface of mannitol. The release of GSH from F1 and F5 formulations was prolonged until 24 h and pH independent. The total antioxidant capacity of GSH was concentration-dependent and maintained after formulation of GSH proliposomes. Circular dichroism spectroscopy confirmed that the molecular structure of GSH was maintained in the proliposome formulations. GSH proliposomes exhibited no significant changes in particle size and zeta potential for 4 weeks. An oral bioavailability study in rats revealed that F5 exhibited 1.05-, 1.08-, and 1.11-fold higher bioavailability than F1, commercial capsule formulation, and pure GSH, respectively. In conclusion, the prepared GSH proliposomes enhanced the poor bioavailability of GSH and prolonged its duration of action.

## Introduction

Usage of conventional chemical substances is limited when the treatment or disease condition has complex mechanisms (Ahmad et al., 2017). For these unmet needs, development of biopharmaceuticals using *in vivo* mechanisms was initiated and soon became the core technology of drug development (Gavrilescu & Chisti, 2005). Among them, development of peptide drugs, which account for ∼10% of biopharmaceuticals, is growing at a rapid rate of 7.7% yearly, and the annual global sales of these drugs are estimated to reach $25.4 billion by 2018 (Ozer & Chilkoti, 2017). Peptide drugs have advantages of high potency, efficacy, stability, and selectivity. However, they also have disadvantages of short half-life, low cell-membrane permeability, and poor oral bioavailability due to gastric and enzymatic degradation (Ahmad et al., 2017; Mohtashamian & Boddohi, 2017).

l-Glutathione (GSH) is a water-soluble tripeptide composed of amino acids such as glutamine, cysteine, and glycine (Townsend et al., 2003). It is the most abundant intracellular low-molecular-weight peptide containing a thiol group (Meng et al., 2009). The thiol group of cysteine in GSH is a potent reducing agent that can perform several important cellular functions, such as metabolism, catalysis, and transport. GSH exhibits diverse physiological roles, such as antioxidant defense, phagocytosis modulation, and reactive oxygen species- scavenging (Bilzer & Lauterburg, 1991). GSH deficiency is responsible for the development of many diseases including kwashiorkor, seizure, Alzheimer’s disease, and Parkinson’s disease (Naji-Tabasi et al., 2017). In Parkinson’s disease, the amount of early GSH is in fact reduced by 40%, and only 2% of its normal state is available (Sian et al., 1994).

Despite these advantages, the low bioavailability and stability of GSH limit its therapeutic application. Peptide drugs including GSH undergo chemical changes and hydrolysis in the gastrointestinal tract after oral administration. Therefore, drug delivery systems for oral administration of GSH are required for it to withstand gastrointestinal environment. Over the past several years, numerous studies have attempted to develop oral formulations of GSH such as chitosan (CS) (Rotar et al., 2014), CS/cyclodextrin (Trapani et al., 2010), and Eudragit® RS 100/cyclodextrin (Lopedota et al., 2009) nanoparticles, as well as mucoadhesive polymer-based film formulations (Chen et al., 2015). However, these previous studies have shown limitations in providing pharmacokinetic data from animal studies. Liposomes are used as carriers for oral delivery of small-molecule drugs, particularly peptides and proteins (Allen & Cullis, 2013). Liposomes are composed of a phospholipid bilayer structure surrounding an aqueous core. Bilayer vesicles are formed through hydrophilic/hydrophobic interactions between lipid-lipid and lipid-water molecules when energy is transferred by, for example, sonication and vortexing. Such properties enable liposomes to encapsulate both lipophilic and hydrophilic drugs. In addition, liposomes can improve drug bioavailability because they have a similar structure to that of cell membranes. In addition, liposomes could be used for mucosal delivery of peptides and proteins (Eloy et al., 2014). Despite their advantages, liposomes also have several stability problems, such as aggregation, drug leakage, sedimentation, and fusion, as well as phospholipid hydrolysis, oxidation, or both.

Considering the stability problems of liposomes, an effective alternative is proliposomes, a formulation in which liquid liposomes are transformed into a solid state (Payne et al., 1986). Proliposomes contain water-soluble carriers coated with phospholipid, and they are dry, free-flowing powders that can be hydrated rapidly into liposomes in physiological fluid. Proliposomes are considered a promising product that provides the convenience of transportation and storage (Xiao et al., 2006). In addition, although liposomes are mainly used as an injection, proliposomes have been used as an oral delivery system for various drugs, such as silymarin (Xiao et al., 2006), indomethacin (Katare et al., 1991), isradipine (Bobbala & Veerareddy, 2012), resveratrol (Basavaraj & Betageri, 2014), simvastatin (Zhang et al., 2010), celecoxib (Nasr, 2010), fenofibrate (Chen et al., 2009), ginkgo biloba extract (Zheng et al., 2015), cyclosporine A (Shah et al., 2006), and salmon calcitonin (Song et al., 2005).

The main purpose of this study was to develop and evaluate proliposomes to increase the oral bioavailability of the peptide drug, GSH. Proliposome formulations were prepared using the wet granulation method, and then characterized for particle size, zeta potential, entrapment efficiency (EE), and *in vitro* drug release. The solid-state of the optimized proliposomes was characterized using differential scanning calorimetry (DSC), scanning electron microscopy (SEM), x-ray diffraction (XRD), and FT-IR spectrometry. The 3-(4,5-dimethylthiazol-2-yl)-2,5-diphenyltetrazolium bromide (MTT) assay was performed to determine the cytotoxicity of GSH and proliposomes. Total antioxidant capacity was investigated and circular dichroism study confirmed that the molecular structure of GSH was stable during proliposome preparation. Stability of GSH proliposomes was evaluated based on particle size, polydispersity index (PDI), and zeta potential of the reconstituted liposomes after storage for 4 weeks. In addition, animal studies were performed to assess the oral bioavailability of GSH.

## Materials and methods

### Materials

Reduced GSH, l-α-phosphatidylcholine (PC), cholesterol (Chol), chitosan (CS; low-molecular-weight, 75–85% deacetylation), 5,5′-dithio-bis(2-nitrobenzoic acid), 3-(4,5-dimethylthiazol-2-yl)-2,5-diphenyltetrazolium bromide (MTT), and sodium phosphate dibasic were purchased from Sigma-Aldrich (St. Louis, MO). 3β-[N-(N′,N′-dimethyl aminoethane)-carbamoyl] cholesterol hydrochloride (DC-Chol) was purchased from Avanti Polar Lipids, Inc. (Alabaster, AL). KB cells were purchased from the Korean Cell Line Bank (KCLB) (Seoul, Korea). Fetal bovine serum (FBS), antibiotics, Dulbecco’s modified Eagle medium (DMEM), and phosphate-buffered saline (PBS) were obtained from Hyclone (Logan, UT). Glutathione assay kit and total antioxidant capacity assay kit were purchased from Biomax (Seoul, Korea). The commercial product Evathione^®^ was purchased form CHO-A PHARM (Seoul, Korea). All solvents were purchased from Samchun Chemical (Pyeongtaek, Korea). All other chemicals were commercial products of analytical- or reagent-grade and used without further purification.

### Preparation of GSH-loaded proliposomes

**Scheme 1.**
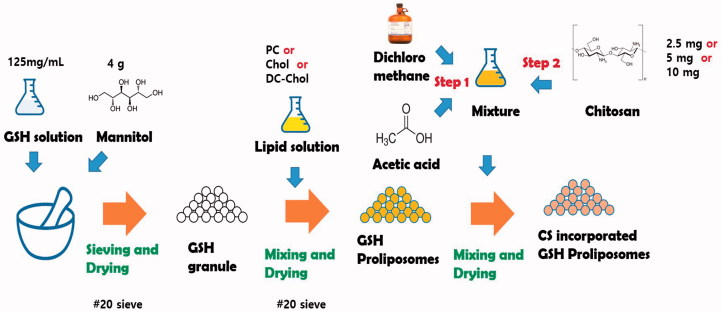
Schematic illustrations for preparation of GSH proliposome.

GSH-loaded proliposomes were prepared according to the granule method of Tantisripreecha et al. (Tantisripreecha et al., 2012) with minor modifications. Using the results of preliminary experiments as a basis, drug to lipid ratio and amount and type of carrier were fixed to achieve the desired particle size, EE, and solubility for oral delivery of the final proliposome formulation. Briefly, 500 mg GSH was dissolved in 4 mL distilled water (DW) and a 3-mL aliquot of the GSH solution was introduced to 4 g mannitol while gently mixed (Scheme 1). The mixture was dried in an oven at 50 °C for 30 min and subsequently sieved through a 20-mesh sieve. Next, PC, Chol, and DC-Chol were weighed to the amounts shown in Table S1 and dissolved in 4 mL dichloromethane. The lipid solution was then poured into the granules and placed in an oven at 50 °C for 2 h to evaporate the organic solvent. The obtained granules were kept at 4 °C before use.

To positively charge the surface of the proliposomes, CS-incorporated proliposomes were also prepared. Briefly, 10 mL dichloromethane and 0.3 mL acetic acid were mixed. CS was weighed to the amounts shown in Table S1 and added to the solvent mixture (0.25%, 0.5%, and 1% w/v). Next, the CS solution was mixed on a magnetic stirrer for 12 h in a tightly closed container. Subsequently, 1 mL of the CS solution was poured into 1 g of proliposomes while gently stirred for 15 min under a fume hood to evaporate the organic solvent. The finished CS-proliposome granules were then dried in an oven at 50 °C for 30 min and the obtained granules were stored at 4 °C before use.

### Characterization of GSH-loaded proliposomes

#### Particle size and zeta potential

Proliposomes (20 mg) were mixed with 10 mL DW and dispersed using probe-type sonicator (KFS-300N ultrasonic processor; Korea Process Technology, Seoul, Korea) at 200 W for 2 min to obtain homogeneous particles. The particle size and size distribution of the reconstituted liposomes were measured using a Zetasizer Nano S90 (Malvern Instruments, Malvern, UK). The zeta potential of the reconstituted liposomes was measured using a Zetasizer Nano Z (Malvern Instruments).

#### Determination of EE and drug loading (DL) capacity

The EE of proliposomes was determined using the modified ultrafiltration method (Yang et al., 2015). Briefly, 20 mg of proliposomes was reconstituted with 10 mL DW and vigorously vortexed at 20 °C for 30 s. The encapsulation and loading efficiency of GSH in the liposomes reconstituted from proliposomal granules were determined by analyzing the filtrate obtained through centrifugation of liposomal suspension using 3,000 MWCO ultracentrifuge tubes (Millipore, Carrigtwohill, Co. Cork, Ireland) at 10,000 rpm for 40 min. The concentration of unentrapped GSH was evaluated following the Ellman’s reagent [5,5′-dithio-bis-(2-nitrobenzoic acid); DTNB] protocol. Briefly, 15 μL of 0.3 M sodium hydrogen phosphate (Na_2_HPO_4_) was added to 60 μL of the filtered solution, followed immediately by 45 μL DTNB. Next, the mixture was stirred for 1 min at room temperature and finally measured for GSH using an ultraviolet (UV) spectrometer infinite M200 PRO (TECAN, Switzerland) at 412 nm. Drug loading (DL; %) capacity, which is the amount of drug encapsulated in the liposome, was also calculated. The EE (%) and DL (%) of GSH were calculated using the equations below.

$$\text{EE }\left(\%\right)=\;\frac{A-B}{A}\times 100$$

$$\text{DL }\left(\%\right)=\;\frac{A-B}{C}\times 100$$

Where A is the total amount of GSH added, B is the unentrapped GSH, and C is the total amount of lipid actually used relative to the yield.

#### Morphology

The morphology of the proliposomes was observed using a LYRA 3 XMU scanning electron microscope (TESCAN, Brno, Republic of Czech). Proliposome powders were subsequently dropped onto glass coverslips, dried, and then evaluated using SEM. Next, the glass coverslip was attached with a carbon tape onto an SEM grid and coated with Pt for 70 s. Mannitol was measured using the same method to investigate its effect on the proliposomal surface.

The morphology of the liposomes was also measured after dispersion. Briefly, a drop of the water-diluted liposomes suspension (0.05 mg/mL) was placed on a 200-mesh formvar copper grid (TABB Laboratories Equipment, Berks, UK) and allowed to adsorb; next, the surplus was removed using filter paper. A drop of a 2% (w/v) aqueous solution of uranyl acetate was added and left in contact with the sample for 5 min. Surplus water was then removed, and the sample was dried at 20 °C. Subsequently, the vesicles were imaged using a transmission electron microscope (TEM) operating at an acceleration voltage of 200 kV (JEM-2100F; JEOL, Peabody, MA). Energy dispersive X-ray spectrometry (EDS) was also conducted using a JEM-2100F field emission electron microscope (JEOL) and images were acquired at 200 kV.

#### DSC analysis

DSC analysis was performed using a DSC 1differentail scanning calorimeter (Mettler Toledo, Barcelona, Spain). The samples were GSH, PC, Chol, DC-Chol, mannitol, proliposomes, and physical mixtures (PMs). PMs were prepared with the same ratio of components as those used to prepare proliposomes. The temperature range was 25 °C–250 °C and the heating rate was 10 °C/min (Manca et al., 2012).

#### FT-IR spectrometry analysis

FT-IR was performed using a VERTEX 80v spectrometer (Bruker, Bremen, Germany). The samples used for FTIR were the same as those used for DSC. Samples were scanned 64 times at a scanning range of 4000 cm^−1^–600 cm^−1^ (Gregorio-Jauregui et al., 2012).

#### XRD analysis

The crystal structures of all samples were evaluated using an X-Pert PRO MPD X-ray diffractometer (Rigaku International Corporation, Tokyo, Japan) with CuKα radiation (40 kV and 40 mA). The samples used for XRD were the same as those used for DSC. XRD patterns were analyzed using the Jade 5.0 software at a range of 5°–70° (Vyas et al., 2010; Choi et al., 2016).

#### *In vitro* drug release study

The release of GSH from proliposomes was measured using a dialysis bag (Karn et al., 2014; Chen et al., 2016). Briefly, proliposomes at an amount corresponding to 3.5 mg GSH was reconstituted with 2 mL DW and then the solution was loaded into a dialysis bag [molecular weight cutoff (MWCO)=25 kDa]. The dialysis bag was immersed into 100 mL release medium (pH 1.2 and 6.8) and stirred at 37 °C at a speed of 100 rpm. Next, 1 mL of the medium was collected at the predetermined time points (1, 2, 4, 8, 12, and 24 h) and replaced with an equal volume of fresh medium. Total GSH concentration in the release medium was measured using a GSH assay kit (Biomax, Seoul, Korea).

#### Cell viability

Cell viability was evaluated using the MTT assay. Briefly, KB cells were seeded in 96-well plates (Nunclon Delta Surface, Thermo Fisher, Beijing, China) at a density of 5 × 10^4^ cells/well and subsequently incubated for 24 h. Medium in each well was replaced with various concentrations of reconstituted liposomes and GSH, and further incubated for 24 h. Next, the wells were washed, and 20 μL MTT solution (10 mg MTT in 2 mL PBS mixed with 10 mL medium) was added to each well and the cells were incubated for 4 h. The MTT solution was removed, and 100 μL of dimethyl sulfoxide was added to the wells. The plate was shaken using a Laboshaker R100 shaker (Labogene, Allerød, Denmark) for 30 min before UV absorbance was measured at 562 nm.

#### *In vitro* total antioxidant capacity study

The total antioxidant capacity of GSH and proliposomes was evaluated using a TAC assay kit (Biomax). Briefly, GSH and proliposomes were dissolved in PBS to prepare GSH solutions of various concentrations (0.025, 0.05, and 0.1 mg/mL). Then, 100 μL of each solution was added to 100 μL copper reagent and reaction buffer. After the reaction was run at room temperature for 30 min, 120 μL samples were measured using an infinite M200 PRO UV spectrometer (TECAN, Männedorf, Switzerland) at 450 nm.

#### Circular dichroism spectroscopy

Circular dichroism measurements of GSH and the GSH proliposomes were performed using a Jasco-815 150-L spectrometer (Jasco International, Tokyo, Japan) with quartz cuvettes at a path length of 1 mm at an ambient temperature. Data were collected every 0.5 nm at a bandwidth and rate of 2.0 nm and 20 nm/min, respectively, with an average of over three scans.

#### Pharmacokinetics study

Male Sprague-Dawley rats, aged 6 weeks and weighing 160–170 g, were purchased from Samtako (Osan, Korea). When the rats were 7 weeks old, they were fasted overnight (14–18 h) and divided into four groups of six animals each for the *in vivo* bioavailability study. Groups A and B received pure GSH solution and commercial GSH product at a dose of 300 mg/kg GSH, respectively, whereas groups C and D received reconstituted proliposomes, F1 and F5, at a dose equivalent of 300 mg/kg GSH. The commercial capsule formulation contained 50 mg GSH per unit. However, for pharmacokinetic evaluation, high-dose GSH was orally administered to rats because the LD50 of GSH is very high (e.g. oral LD_50_=5000 mg/kg in mouse). Approximately 0.5 mL blood samples were collected from the fossa orbitalis vein at 0, 1, 2, 4, 8, 12, and 24 h post-dosing. Then, the blood samples were centrifuged at 15,000 rpm for 12 min to obtain plasma samples, which were stored at -20 °C until the GSH quantification study. Because GSH is an endogenous substance, plasma was used without protein removal process using an organic solvent. Samples were analyzed using the same method described in the previous section (Determination of EE and drug loading capacity) (Yang et al., 2015). On the basis of GSH oxidation by DTNB to form the yellow derivative 5′-thio-2-nitrobenzoic acid (TNB), GSH was quantitated using a UV spectrometer at 412 nm (Rahman et al., 2006). The study protocol complied with the institutional guidelines for the care and use of laboratory animals and was approved by the ethics committee of Chungnam National University (No. CNU-00970).

#### Stability of GSH proliposomes

To evaluate proliposome stability, we measured particle size, PDI, and zeta potential at the predetermined times (0, 1, 2, 3, and 4 weeks) (Bai et al., 2011; Srisuk et al., 2012). Samples were stored at 4 °C before measurements.

### Statistical analysis

All data were expressed as the means ± standard deviation (SD). The data were analyzed using the SigmaPlot software (version 12.0; Systat Software Inc., San Jose, CA). Statistical analysis between different groups was performed using the *t*-test, and *p* < .05 indicated a significant difference.

## Results and discussion

### Preparation of GSH proliposomes

To study the effect of components on the physicochemical characteristics of proliposomes, the PC:Chol ratio and concentration of charged lipid was compared. CS was used to verify the effect of the surface charge. Using data from the preliminary studies, the drug:lipid ratio and amount of carrier were fixed based on the liposome formulation possibility, particle size, and encapsulation efficiency. To address the physical and chemical instability of liposomes, proliposomes were developed using a method that, when needed, can produce liposomes quickly without excessive manipulation (Muneer et al., 2017). Several methods have been reported for preparing proliposomes, including film deposition on the carrier, spray-drying, fluidized-bed, supercritical anti-solvent, and granulation (Patel et al., 2017). The granulation method was chosen for this study because of its simplicity and efficiency, and it was modified to prepare GSH proliposomes.

### Characterization of GSH proliposome

#### Particle size, zeta potential, and entrapment efficiency of proliposomes

The particle size and zeta potential of GSH proliposomes are shown in Table S2. The particle sizes of F1, F2, and F3 (167.8 ± 0.9 nm, 260.2 ± 4.9 nm, and 677.4 ± 14.4 nm, respectively), led us to deduce that particle size increased along with Chol to PC ratio. PDI also showed a similar pattern. Because it was reported that liposomes of 100–200 nm size could enhance the oral bioavailability of incorporated ingredients (Chono et al., 2007; Ong et al., 2016), F1 was selected for further optimization of formulation. The addition of DC-Chol to the F1 formulation did not alter particle size as observed in F4, F5, and F6. When CS was added, the particle size was almost twice as large as that of F1 (320–330 nm).

To increase the efficiency of proliposomes, we attempted to positively charge the particles while maintaining the composition ratio of the F1 formulation. Cationic surfactants can increase the permeation of nanoparticles, but it can cause toxicity and interfere with circulation in the body (Pillai et al., 2015; Salatin et al., 2015; Wu et al., 2018). Therefore, DC-Chol and CS were selected to positively charge the proliposomes. Moreover, CS has been reported to be mucoadhesive and can increase intestinal absorption rate (Zhang et al., 2015b; Kang et al., 2017). As the ratio of DC-Chol increased, zeta potential increased (Table S2). When CS was added to the F1 formulation, the zeta potential of proliposomes positively increased from −8.1 mV (F1) to 23.0 mV (F7), 26.5 mV (F8), and 29.4 mV (F9) according to the amount of CS (Table S2).

The EE of GSH in proliposomes was determined because EE is a critical factor in determining the therapeutic effect and dose regimen of drugs. As the ratio of Chol increased, entrapment efficiency decreased; the EE of F1, F2, and F3 were 58.6 ± 0.7%, 51.3 ± 0.6%, and 42.9 ± 0.4%, respectively. It is known that Chol prevents the loss of hydrophilic materials and stabilizes lipid bilayer by decreasing the rotational freedom of phospholipid hydrocarbon chains (Manojlovic et al., 2008). However, in our studies, the addition of Chol to liposomes decreased the EE of GSH because the space was too narrow to allow GSH incorporation. Hydrophilic compounds such as GSH are incorporated into the aqueous core of liposomes (Han et al., 2018). Thus, the process is associated with the creation of high-volume internal aqueous phase, resulting in low EE values (Feng et al., 2016). The EE of proliposomes containing DC-Chol was more than 50%. However, when CS was added the EE decreased to 35% or less (Table S2). Considering the particle size, zeta potential, and EE of the proliposomes, it was desirable to add DC-Chol instead of CS. Then, further experiments were conducted by selecting F1 and F5 formulations.

#### Morphology of GSH proliposome and reconstituted liposomes

**Figure 1.**
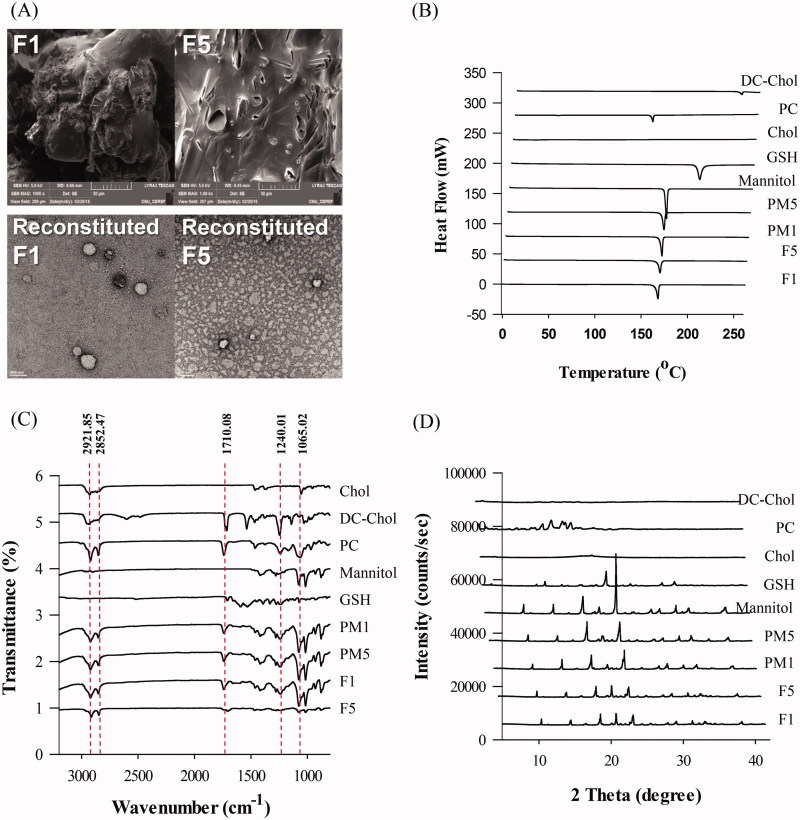
Physicochemical characterization of proliposomes. (A) (upper) SEM image of proliposomes (F1 and F5) and (lower) TEM image of reconstituted liposomes from F1 and F5, (B) Differential scanning calorimetry curves of GSH, PC, Chol, DC-Chol, mannitol, proliposomes, and PM, (C) Fourier transform infrared spectra of GSH, PC, Chol, DC-Chol, mannitol, proliposomes and PM, and (D) X-ray diffractometry spectra of GSH, PC, Chol, DC-Chol, mannitol, proliposomes, and PM.

The physical state and surface characteristics of proliposomes were evaluated using SEM, and as shown in Figure 1(A) (upper), mannitol evidently existed in the typical crystalline form. The needle-shaped crystalline form of mannitol was undecipherable because of the deposition of the phospholipids on the surface. The thin lipid film coating on the surface of the mannitol granules ensured complete hydration of the phospholipid, leading to liposomal dispersion following contact with a medium (Tantisripreecha et al. 2012).

TEM analysis confirmed the presence of liposomes in the reconstituted proliposomes and revealed their morphological properties. TEM images of the reconstituted liposomes (Figure 1(A) lower) showed a dense and spherical structure, as well as the formation of liposomes below 200 nm, which is consistent with the dynamic light scattering results.

EDS mapping (Figure S1(A)) showed that uranyl acetate was more abundant in the aqueous solution than in the liposomes. Uranyl acetate was detected using additional elemental indexing (labeling) of the spectrum (Figure S1(B)). Because the liposomes were composed of a hydrophobic phospholipid bilayer, they had a low permeability for uranyl acetate. The EDS mapping data also confirmed the spherical shape of the liposomes.

#### DSC analysis

DSC analysis evaluated the physical state and thermal properties of the drug and carrier in the formulation. The DSC profiles of GSH, PC, Chol, DC-Chol, mannitol, proliposomes, and PMs are shown in Figure 1(B). The proliposome formulations F1 and F5 showed no characteristic exothermic peaks for GSH at 200.45 °C. However, mannitol peaks at 166.01 °C and 166.29 °C remained, which suggested that the mannitol coating on their surfaces was incompletely formed. The enthalpy (ΔH) of the phase transition temperature of mannitol was 309.76 J/g. The proliposomal formulation of mannitol significantly affected the ΔH of mannitol. Figure 1(D) also shows that mannitol was present in the proliposomes. The mannitol-containing proliposomes F1 and F5 decreased the ΔH of mannitol from 201.01 J/g and 199.77 J/g, respectively. The phase transition peak of GSH eventually disappeared. These results indicated that lipid incorporation contributed to the conversion of mannitol and GSH in the proliposomes from the crystalline to amorphous form.

#### FT-IR spectrometry

FT-IR analysis was performed to investigate possible interactions between GSH and the excipients. The FT-IR spectra of GSH, PC, Chol, DC-Chol, mannitol, proliposomes, and PMs are shown in Figure 1(C). Structural information about the lipid bilayer interior was determined from the vibrational frequency of the symmetric CH_2_ stretching (Bai et al., 2011). The FT-IR spectra of PC at 2921.85 and 2852.47 cm^−1^ corresponding to the stretching vibration absorption of CH_2_ was not changed in the PMs and proliposomes. We discovered that the granulation method used did not affect the interior of the bilayer. The other characteristic peaks of PC appeared at 1740 cm^−1^ for C = O stretching vibrations and at 1240.01 and 1065.02 cm^−1^ for PO_3_^−1^ stretching vibrations. There were no significant changes in the CH_2_ and PO_3_^−1^ peaks of PMs and F1. However, by comparing the FT-IR spectra of F5 and PC, we clearly showed that there was a significant interaction between GSH and the lipid components and mannitol in the proliposomes.

#### X-ray diffraction study

XRD diffractograms are shown in Figure 1(D) and those of pure crystalline mannitol indicated sharp characteristic peaks at diffraction angles (2 Theta) of 10.44, 14.6, 16.7, 18.7, 20.9, 23.3, 25.8, 28.2, 31.7, 33.35, and 38.68, which are similar to the peaks reported in the literature. For proliposome powder, the XRD patterns of GSH, lipid, and mannitol were dependent on the formulation compositions (Rojanarat et al., 2011). The ratio of mannitol in F1 and F5 was higher than that in other formulations, which could have significantly affected the remaining mannitol peaks. However, the results showed that the intensities of the mannitol peaks were reduced, indicating good correlation with the DSC results.

#### *In vitro* release study

**Figure 2.**
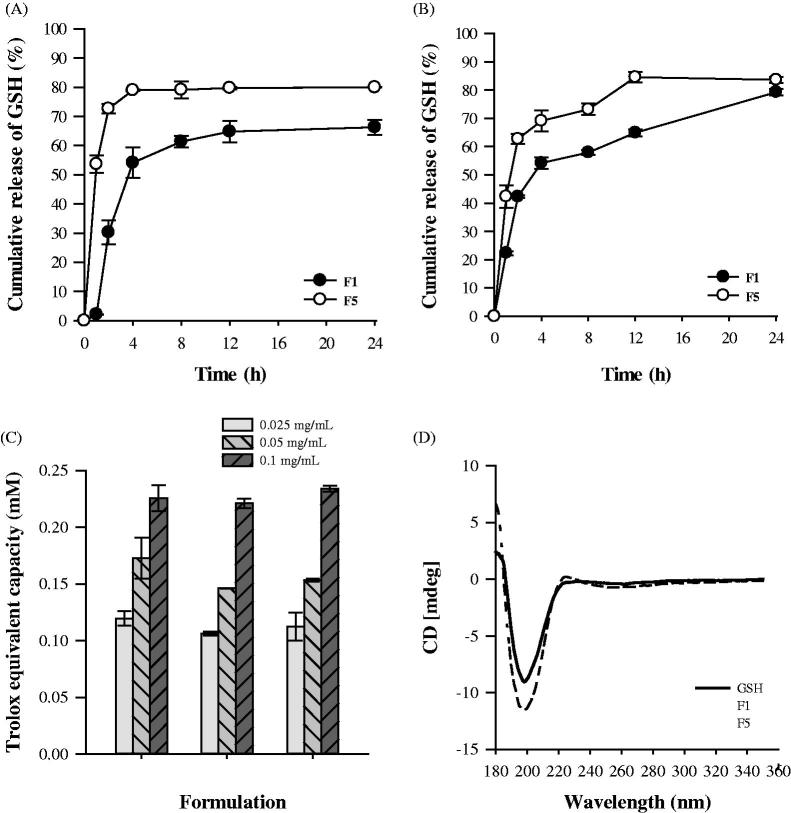
Release profiles of GSH from rehydrated proliposomes at pH 1.2 (A) and pH 6.8 (B) (*n* = 3, mean ± SD), (C) Total antioxidant capacity of pure GSH and GSH proliposomes (*n* = 3, mean ± SD), and (D) Circular dichroism spectra of GSH and proliposomes.

The GSH release profiles of rehydrated proliposomes were observed at pH 1.2 and 6.8 to perform oral delivery of GSH-loaded proliposomes (Figure 2(A,B)). The release of GSH from the rehydrated F1 liposomes was significantly lower than that from F5 at both pH 1.2 and 6.8. A high absolute zeta potential value could cause strong repellent forces among particles to prevent aggregation (Yousefi et al., 2009). However, because the release profiles were similar, the rehydrated liposomes were considered to be non-pH-responsive.

At pH 1.2 and 6.8, ∼73% and 62% of GSH from the rehydrated F5 proliposomes was released at 2 h, and the release amount peaked at 4 and 12 h, respectively. It is well known that the carboxyl and amino groups of GSH are sensitive to pH, which could have significantly influenced its release from the rehydrated proliposomes. The amino groups of GSH can be fully protonated and exhibit high positive charges at pH 1.2. In the positively charged F5, strong electrostatic intermolecular repulsion could have enhanced the leakage of GSH. However, carboxyl groups have negative charges at pH 6.8, and therefore, the electrostatic attractive force reduced the release of GSH.

#### Cell viability

After sample incubation, cellular toxicities were measured using the MTT assay at the GSH concentrations of 0.01, 0.1, 1, 3, 10, and 30 µg/mL (Figure S2). GSH was originally nontoxic, with cell viability reaching 90% at almost all the tested concentrations. Cell viability did not change in all formulations when GSH concentration increased from 0.01 to 30 µg/mL.

#### *In vitro* total antioxidant capacity study

Antioxidants play an important role in preventing the formation of potentially toxic oxidants. GSH and its associated enzymes form a major antioxidant defense system in all cells (Zeevalk et al., 2010). Other antioxidants depend on their reducing power, but Trolox is used as a standard antioxidant, and all other antioxidants are measured in Trolox equivalents. The total antioxidant capacity assay kit used is based on the principle that most antioxidants are in the reduced form, and the oxidation of Cu^2+^ to Cu^+^ by antioxidants is determined by measuring the absorbance at 450 nm using a colorimetric method.

The total antioxidant capacity of pure GSH and GSH proliposomes is presented in Figure 2(C). The Trolox equivalent capacity of GSH and proliposomes was similar; therefore, encapsulation in the liposomes did not interfere with the antioxidant capacity of GSH. In addition, the antioxidant capacity of GSH proliposomes was confirmed by the increasing activity observed with increasing GSH concentration.

#### Circular dichroism spectroscopy

Chirality is a unique phenomenon in nature, and essential physiological molecules, such as proteins and nucleic acids, are chiral. All other amino acids, excluding glycine in proteins, are l-enantiomers. Currently, chirality has contributed to great advances in nanoparticle development (Zhang et al., 2015). Figure 2(D) shows the circular dichroism of GSH and GSH proliposomes. The optical activity of GSH was maintained in proliposome formulations. Furthermore, the molecular structure of GSH was not damaged during the preparation of GSH proliposomes using the granulation method.

#### Pharmacokinetics study

**Figure 3.**
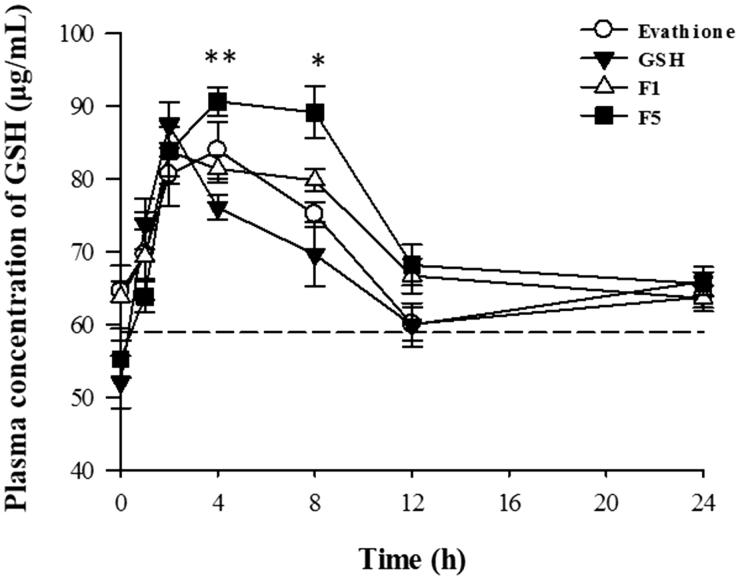
Rat plasma concentrations of GSH as a function of time following oral administration of GSH, commercial product and GSH-proliposomes (*n* = 6, mean ± SD).

A comparative pharmacokinetic study of GSH, commercial product, and proliposomes was carried out. GSH concentrations were analyzed in rat plasma after oral administration and the mean plasma concentration-time profiles are shown in Figure 3. The main pharmacokinetic parameters of plasma GSH concentration versus time profiles are listed in Table 1.

**Table 1.** Pharmacokinetic parameters of pure glutathione (GSH), commercial product (Evathione^®^), and proliposomes (F1, F5) after oral administration in rats (*n* = 6, mean ± S.D).

	GSH	Eva	F1	F5
AUC_last_ (h**·**μg/mL)	1610 ± 79	1637 ± 94	1703 ± 68	1782 ± 92*
C_max_**(**μg/mL)	87.7 ± 6.7	86.6 ± 9.6	88.2 ± 3.9	92.9 ± 6.6
T_max_ (h)	2.3 ± 0.8	3.3 ± 0.9	3.2 ± 2.3	4.3 ± 1.8*

In all analyses, *p* < .05.

* Statistical significance.

In Figure 3, all spots were higher than the baseline plasma GSH concentration (58.9 μg/mL). All F5 spots at 4 h after oral administration were higher than those of other formulations. In particular, at 4 and 8 h, plasma GSH concentration was significantly higher in the group orally administered F5 than in those administered pure GSH (4 h, *p* < .005; 8 h, *p* < .01). The overall average values of F5 are slightly higher than those of commercial products, although there were no statistically significant differences.

The maximum concentration (C_max_) values of pure GSH, commercial product, F1, and F5 were 87.7 ± 6.66, 86.6 ± 9.6, 88.2 ± 3.9, and 92.9 ± 6.6 μg/mL, respectively. The C_max_ of F5 increased to 105.9% compared to that of pure GSH. The area under the concentration-time curve up to the last time point (AUC_last_) values of pure GSH, commercial product, F1, and F5 were 1610 ± 79, 1637 ± 94, 1703 ± 68, and 1782 ± 92 h·μg/mL, respectively. The AUC_last_ of F5 increased to 110.6% compared to that of pure GSH, which was significantly different from that of GSH; however, that of F1 was not significantly different from that of GSH. The time to achieve C_max_ (T_max_) of pure GSH, commercial product, F1, and F5 was 2.3 ± 0.75, 3.3 ± 0.9, 3.2 ± 2.3, and 4.3 ± 1.8 h, respectively. The T_max_ of F5 was 1.86 times higher than that of pure GSH, and this effect was significantly different from that of GSH, but that of F1 was not different from that of GSH. This confirms that the release of GSH from F5 was more extended than that from F1. The improvement in the oral bioavailability of GSH could be attributed to the extended release due to proliposomes, which increased the amount of drug available for gastrointestinal absorption. In addition, the liposomes adhered to the epithelial cell membrane (Zheng et al., 2015).

#### Stability test

**Figure 4.**
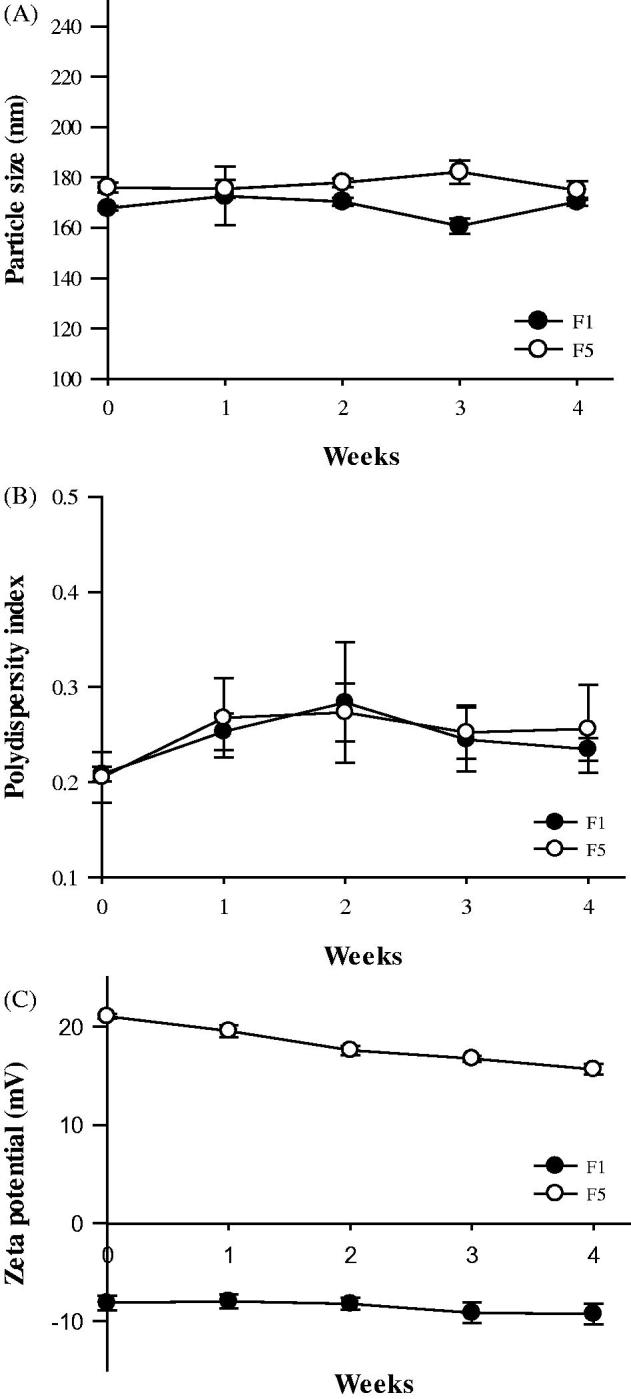
Stability of GSH-loaded proliposomes. The particle size (A), PDI (B), and zeta potential (C) of the proliposomes was investigated for 4 weeks (*n* = 3, mean ± SD).

The stability of F1 and F5 based on particle size, PDI, and zeta potential is shown in Figure 4. The initial particle sizes of F1 and F5 were 167.8 ± 0.9 and 175.9 ± 1.95 nm, respectively, which changed to 170.4 ± 4.57 and 174.9 ± 3.7 nm, respectively, after 4 weeks of storage at 4 °C. The PDI values of F1 and F5 were changed from 0.21 and 0.20 to 0.23 and 0.26, respectively, whereas their corresponding zeta potentials were changed from −8.1 and 21.1 mV to −9.3 and 15.6 mV, respectively. Therefore, no significant changes in stability were observed in this study.

## Conclusion

In this study, a novel proliposomal oral delivery system for GSH was successfully prepared using the wet granulation method. The particle size and zeta potential of the reconstituted liposomes depended on the amount of the lipids. F1 and F5 showed suitable particle sizes and zeta potentials, as well as good stability after a 4-week storage. The formulations showed sustained release profiles at both pH 1.2 and 6.8. The total antioxidant capacity and circular dichroism of GSH was maintained in the GSH proliposomes. As shown in the cytotoxicity test, 24 h of exposure of GSH and GSH proliposomes were nontoxic to KB cells. In the pharmacokinetic study, F1 and F5 were superior to GSH and commercial formulation in terms of oral bioavailability. In conclusion, the proliposomes could improve the poor oral bioavailability of glutathione.

## Supplementary Material

Byeon_JC_Supporting_Information_20181112________.docx
