# Supplementary material for: Design of novel proliposome formulation for antioxidant peptide, glutathione with enhanced oral bioavailability and stability
Source: Drug Deliv. 2019 Mar 7;26(1):216–25. doi: 10.1080/10717544.2018.1551441 (PMC6407602; doi:10.1080/10717544.2018.1551441)
Supplement: Byeon_JC_Supporting_Information_20181112________.docx [file IDRD_A_1551441_SM9590.docx]

**Supporting information**

**Design of novel proliposome formulation for antioxidant peptide, glutathione with enhanced oral bioavailability and stability**

Jong Chan Byeon^a,1^, Sang-Eun Lee^a,1^, Tae-Hyeon Kim^a^, Jung Bin Ahn^a^, Dong-Hyun Kim^a^, Jin-Seok Choi^b^, Jeong-Sook Park^a,^*

^a^ College of Pharmacy and Institute of Drug Research and Development, Chungnam National University, 99 Daehak-ro, Yuseong-gu, Daejeon 34134, South Korea

^b^ Department of Medical Management, Chodang University, 380 Muan-ro, Muan-eup, Muan-gun, Jeollanam-do 58530, South Korea

^1^ Both authors contributed equally to this work.

*To whom correspondence should be addressed.

Jeong-Sook Park, Ph.D.

College of Pharmacy, Chungnam National University, 99 Daehak-ro, Yuseong-gu, Daejeon 305-764, Republic of Korea

Tel: +82-42-821-5932, Fax: +82-42-823-6566

Email: eicosa@cnu.ac.kr

**Table S1.** Composition of GSH-loaded proliposomes.

| Formulation | F1 | F2 | F3 | F4 | F5 | F6 | F7 | F8 | F9 |
| --- | --- | --- | --- | --- | --- | --- | --- | --- | --- |
| GSH (g) | 0.375 | 0.375 | 0.375 | 0.375 | 0.375 | 0.375 | 0.375 | 0.375 | 0.375 |
| Mannitol (g) | 4 | 4 | 4 | 4 | 4 | 4 | 4 | 4 | 4 |
| PC (g) | 0.8 | 0.5 | 0.2 | 0.8 | 0.8 | 0.8 | 0.8 | 0.8 | 0.8 |
| Chol (g) | 0.2 | 0.5 | 0.8 | 0.2 | 0.2 | 0.2 | 0.2 | 0.2 | 0.2 |
| DC-Chol (mg) | 0 | 0 | 0 | 12.5 | 25 | 50 | 0 | 0 | 0 |
| Chitosan (mg) | 0 | 0 | 0 | 0 | 0 | 0 | 2.5 | 5 | 10 |

**Table S2.** Particle size, PDI, zeta potential and entrapment efficiency (%) of formulation 1-9. Data are expressed as the mean ± S.D (n=3).

| Formulation | Size (nm) | PDI | Zeta potential (mV) | EE (%) | DL (%) |
| --- | --- | --- | --- | --- | --- |
| F1 | 167.8 ± 0.9 | 0.21 | -8.1 ± 0.7 | 58.6 ± 0.7 | 86.9 ± 1.0 |
| F2 | 260.2 ± 4.9 | 0.43 | -14.7 ± 0.1 | 51.3 ± 0.6 | 74.1 ± 0.9 |
| F3 | 677.4 ± 14.4 | 0.73 | -18.6 ± 0.9 | 42.9 ± 0.4 | 63.2 ± 0.5 |
| F4 | 162.8 ± 3.8 | 0.20 | -3.5 ± 0.1 | 50.8 ± 1.2 | 72.3 ± 1.7 |
| F5 | 175.9 ± 2.0 | 0.21 | 21.1 ± 0.2 | 54.7 ± 0.1 | 80.8 ± 0.1 |
| F6 | 141.1 ± 1.9 | 0.20 | 31.4 ± 1.0 | 51.0 ± 1.1 | 74.8 ± 1.6 |
| F7 | 320.1 ± 5.9 | 0.32 | 23.0 ± 0.9 | 35.7 ± 1.4 | 21.0 ± 1.9 |
| F8 | 323.0 ± 3.0 | 0.36 | 26.5 ± 0.3 | 31.1 ± 0.9 | 24.7 ± 1.7 |
| F9 | 330.8 ± 17.0 | 0.31 | 29.4 ± 1.1 | 14.7 ± 0.6 | 20.1 ± 0.1 |

**Fig. S1.** Energy dispersive X-ray spectrometer of liposomes reconstituted from F5. (A) EDS mapping (B) Elemental indexing (labeling) of spectrum.

(A)

(B)

**Fig. S2.** Cell viability incubated with GSH and liposome reconstituted from GSH proliposomes (n=3, mean ±SD).

**Dose of GSH (μg/mL)**

0.01

0.1

30

10

3

1
